# Engineering HOF-Based Mixed-Matrix Membranes for Efficient CO_2_ Separation

**DOI:** 10.1007/s40820-023-01020-w

**Published:** 2023-02-14

**Authors:** Yuhan Wang, Yanxiong Ren, Yu Cao, Xu Liang, Guangwei He, Hanze Ma, Hongliang Dong, Xiao Fang, Fusheng Pan, Zhongyi Jiang

**Affiliations:** 1 Key Laboratory for Green Chemical Technology of Ministry of Education, School of Chemical Engineering and Technology, Tianjin University, Tianjin, 300350 People’s Republic of China; 2 Haihe Laboratory of Sustainable Chemical Transformations, Tianjin, 300192 People’s Republic of China; 3 Guangdong Laboratory of Chemistry and Fine Chemical Industry Jieyang Center, Jieyang, 522000 Guangdong Province People’s Republic of China; 4 Center for High Pressure Science and Technology Advanced Research, Pudong, Shanghai, 201203 People’s Republic of China

**Keywords:** Hydrogen-bonded organic framework, Tunable size, Mixed-matrix membrane, Mixed-bond, Carbon capture

## Abstract

Hydrogen-bonded organic frameworks (HOFs) have emerged as a new class of crystalline porous materials, and their application in membrane technology needs to be explored. Herein, for the first time, we demonstrated the utilization of HOF-based mixed-matrix membrane for CO_2_ separation. HOF-21, a unique metallo-hydrogen-bonded organic framework material, was designed and processed into nanofillers via amine modulator, uniformly dispersing with Pebax polymer. Featured with the mix-bonded framework, HOF-21 possessed moderate pore size of 0.35 nm and displayed excellent stability under humid feed gas. The chemical functions of multiple binding sites and continuous hydrogen-bonded network jointly facilitated the mass transport of CO_2_. The resulting HOF-21 mixed-matrix membrane exhibited a permeability above 750 Barrer, a selectivity of ~ 40 for CO_2_/CH_4_ and ~ 60 for CO_2_/N_2_, surpassing the 2008 Robeson upper bound. This work enlarges the family of mixed-matrix membranes and lays the foundation for HOF membrane development.

**Supplementary Information:**

The online version contains supplementary material available at 10.1007/s40820-023-01020-w.

## Highlights

- For the first time, the hydrogen-bonded organic frameworks (HOFs)-based mixed-matrix membranes were designed for CO_2_ separation
- A size-controllable method for synthesis of HOF-21 nanoparticles was proposed via diethylamine-modulated framework assembly process
- The HOF-21 mixed-matrix membranes exhibited outstanding separation performance with P_CO2_ of ~ 800 Barrer, and S_CO2/CH4_ of ~ 40, S_CO2/N2_ of ~ 60, surpassing the 2008 Robosen upper bound.

**Supplementary Information:**

The online version contains supplementary material available at 10.1007/s40820-023-01020-w.

## Graphical abstract

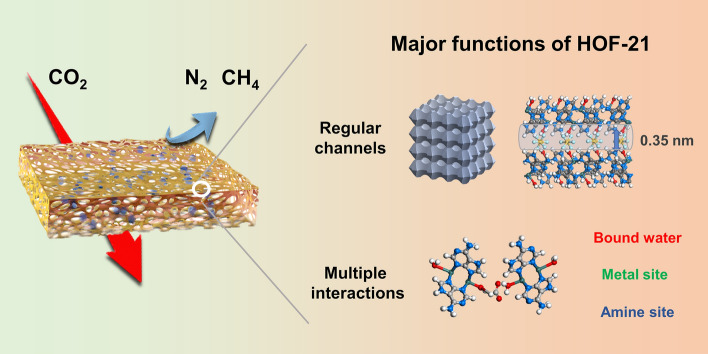

## Introduction

With the advent of carbon neutrality, membrane-based technology has received a burgeoning interest in carbon capture, featured with the energy efficiency, module compactness and operational simplicity [1–5]. Mixed-matrix membrane (MMM) has deemed as an advanced membrane platform, with the merit of deploying distinct separation properties of fillers into processable matrix [6–10]. Along with the emergence of reticular chemistry, porous framework materials have been widely developed into advanced fillers to achieve membrane structure optimization and performance enhancement [11–13].

Hydrogen-bonded organic frameworks (HOFs) have emerged as a new class of organic framework materials with high crystallinity and designable structure [13–15]. Due to the reversible nature of hydrogen bond, the HOF materials could be assembled under mild condition and maintained stable after solvent removal, showing excellent potential for gas separation [16–22]. Recently, Huang et al. incorporated HOF-30 crystals into polyimide matrix to prepare the MMMs [23]. Due to permanent porosity and appropriate pore size of 0.4 nm, HOF-30 significantly improved the gas separation performance, with a high permeability of 428.1 Barrer and H_2_/CH_4_ separation factor of 61.7. Nowadays, HOF-based membrane is still in early development stage, and their applications in many significant separation tasks including carbon capture, alkanes/alkenes separation have not been reported. Compared with the MOF materials, developing HOF into advanced fillers to meet different separation requirements remains to be urgently explored.

Herein, we demonstrated using HOF-based MMM in carbon capture for the first time. HOF-21 ([Cu_2_(ade)_4_(H_2_O)_2_](SiF_6_)_2_) was selected as the filler and incorporated into Pebax polymer to form MMMs. HOF-21 [24, 25] belonged to metallo-hydrogen-bonded organic framework materials and featured a unique mix-bonded framework, possessing the following advantages for CO_2_ separation: (1) Mix-bonds with the coordination bond of Cu-ade and the muti-file hydrogen bond of N–H–F and N–H–N ensured the rigidity and stability under humid feed gas. (2) HOF-21 endowed the membranes with more efficient transport channels and facilitated transport mechanism, by the synergy of bound water, metal ion, biogenic amine molecule and hydrogen-bonded network. (3) Through decoupling the assembly process and the crystallization process, amine modulator could be used for tuning size of HOF-21 particles from micro-to-nanoscale. As expected, the desired separation properties of HOF-21 were transplanted into MMMs, which is verified by the superior CO_2_ separation performance.

## Experimental Section

### Materials

Pebax(R) MH 1657 was purchased from Tianjin Kelaite Co., Ltd. Copper nitrate trihydrate (Cu(NO_3_)_2_.3H_2_O, 99%) was bought from Meryer Co., Ltd. Acetonitrile (CH_3_CN, 99.5%) and diethylamine (99%) were provided by Aladdin Co., Ltd. Adenine (ade, 98%) was obtained from Tianjin Heowns Co., Ltd. Ammonium fluorosilicate ((NH_4_)_2_SiF_6_, 98%) was offered from 9ding Co., Ltd. Anhydrous ethanol (99.7%) and dichloromethane were purchased from Tianjin Kermel Co., Ltd.

### Synthesis of HOF-21 Microcrystals and HOF-21 Nanoparticles.

HOF-21 ([Cu_2_(ade)_4_(H_2_O)_2_](SiF_6_)_2_) microcrystals (HOF-21m) were synthesized according to the previous literature [24]. 0.2 mmol Cu(NO_3_)_2_.3H_2_O and 0.4 mmol ade were dissolved in 10 mL of 1:1 acetonitrile/H_2_O to form Cu-ade solution. Then, 0.2 mmol (NH_4_)_2_SiF_6_ was added into the solvent and the crystal precipitated after 6–24 h. Purple HOF-21 microcrystals were obtained after filtration and washing with water and dichloromethane. HOF-21 nanoparticles (HOF-21n) were obtained via introducing diethylamine to regulate the reaction process. 0.2 mmol (NH_4_)_2_SiF_6_ and a certain amount of diethylamine were added into the Cu-ade solution and reacted for 15 min. The purple HOF-21n was collected by centrifugation and washed by water and dichloromethane. The amount of diethylamine were in the range of 0.1–0.4 mmol, and the optimal value was 0.2 mmol.

### Fabrication of Membranes

The HOF-based MMMs were fabricated by adding the HOF-21 fillers into the Pebax® 1657 polymer matrix based on a solution-casting method. In brief, 7.2 g Pebax pellets were added into a 200-mL mixed solvent (70 wt% ethanol and 30 wt% water) and vigorously stirred for 2 h to obtain the Pebax solution. A certain amount of HOF-21n were added into the Pebax solution and stirred overnight to obtain HOF-21@ Pebax solution. Then, the HOF-21@ Pebax solution casted onto Teflon Petri dishes and then dried under ambient conditions for 24 h, followed by drying in the vacuum oven at 45 °C for another two days. The obtained membrane was denoted as HOF-21@ Pebax-*x*, where *x* represents the *x*% mass fraction of fillers in the membrane. In addition, the Cu(NO_3_)_2_ and Cu(ade)_2_ were also used as the fillers and the related membranes were fabricated via following the similar process.

### Characterization

The chemical compounds and bonding of HOF-21m, HOF-21n and a series of HOF-21@ Pebax membranes were confirmed by Fourier transform infrared spectroscopy (FTIR, Bruker Vertex 70). The solid-state ^13^C nuclear magnetic resonance (NMR) spectrum of HOF-21m and HOF-21n was obtained on a Bruker 600 MHz NMR spectrometer (Jeol Jnw Ecz600r). The surface and cross section morphology of powders and membranes were obtained from scanning electron microscope (SEM, Regulus 8100) with energy disperse spectroscopy (EDS). Thermogravimetric analysis (TGA) of HOF was tested on a thermogravimetric analyzer device (NETZSCH TG 209 F3) from 40 to 800 °C with a heating rate of 10 °C min^−1^ under flowing N_2_ atmosphere. The crystallinity of HOF-21 series was evaluated via X-ray diffraction (XRD, (D8-Focus, CuKα)). The total scattering was carried out with the beam energy of 40 keV at the 13w beamline at SSRF, Shanghai. The *G*(*r*) and *S*(*q*) were calculated using the PDFget3. CO_2_ CH_4_ and N_2_ sorption isotherms at 298 K were measured at gas adsorption instrument (Belsorp-Max apparatus). The surface area and pore size distribution were calculated by GCMC method. The thermodynamics properties of membranes were measured using a differential scanning calorimetry (DSC, Netzsch 200F3). The density functional theory calculations were performed using Dmol3 program. Generalized gradient approximation with Perdew–Burke–Ernzerhof (GGA-PBE) function was used for the exchange–correlation. The SCF convergence for each electronic energy was set as 1.0 × 10^−6^ Ha. The convergence tolerances of energy, force and displacement were set at 1 × 10^−5^ Ha, 0.002 Ha Å^−1^, and 0.005 Å, respectively.

### Test of Membrane Performance

The permeation experiments were conducted based on the Wicke–Kallenbach method, with CO_2_/CH_4_ (30 vol%:70 vol%) and CO_2_/N_2_ (20 vol%:80 vol%) as the binary mixed gas. The gas composition determined by gas chromatography (Agilent 7820B). The feed and sweep gas were humidified by the humidification tanks at 40 °C. In the performance test, the pressure on the upstream side was 2 bar, and the sweep gas pressure on the downstream side was 1 bar.

The gas permeability (*P*, 1 Barrer = 10^−10^ cm^3^ (STP) cm^−1^ s^−1^ cmHg^−1^) and selectivity (*S*) were calculated using Eqs. (1) and (2):

1 $$P = Q*\frac{L}{\Delta p* A}$$

2 $$Si/j = Pi/Pj$$

where *Q* is the volumetric flow rate of gas *i*, *L* is the membrane thickness (cm), Δ*p* is the partial pressure difference of gas across the membrane (bar), and *A* is the effective membrane area (cm^2^).

## Results and Discussion

### Synthesis and Regulation of HOF-21

HOF-21 represents a unique HOF subclass, constructed by the metal-complexes as the building units. Conventional HOF materials commonly use the pure organic linkers as building blocks linked by hydrogen bonds alone, while HOF-21 is constructed by both hydrogen bonds and coordination bonds, called metallized HOFs or metallo-HOFs. Different from the conventional MOF materials, most chemical groups are occupied for the formation of bulk framework and thus have much less chance to participate in the facilitated transport of CO_2_. In contrast, for metallo-HOFs, including HOF-21, more chemical groups are reserved to realize the synergy of framework formation and facilitated transport of CO_2_. Within the HOF-21, Cu^2+^ coordinated with ade to form Cu(ade)_2_ complexes which were self-assembled into a supramolecular ribbon chain and hydrogen-bonded with ${\text{SiF}}_{6}^{2 - }$ and water to construct the target framework. The [Cu_2_(ade)_4_(H_2_O)_2_]^4+^ and ${\text{SiF}}_{6}^{2 - }$formed a two-dimensional layer in ab plane and further hydrogen-bonded linked with ${\text{SiF}}_{6}^{2 - }$ via the hydrogen bonding to construct one-dimensional channels paralleled to the c-axis. The coordination reaction and hydrogen bond assembly were commonly achieved in one pot, but stepwise decoupling was performed to better regulate the reaction process in this work. The solubility of ade is of 0.5 g L^−1^ (20 °C) and the complexation with Cu^2+^ could enhance the dissolution to form homogeneous Cu(ade)_2_ solution. The Cu(ade)_2_ complexes could be obtained by evaporating the solvent and displayed weak crystallinity (Fig. S1). The hydrogen bond assembly was achieved between Cu(ade)_2_ and ${\text{SiF}}_{6}^{2 - }$, with the significantly improved crystallinity. The resulting HOF-21displayed the obvious Bragg peaks, as shown in Fig. S2. It is noteworthy that HOF-21 exhibited good structural stability and could maintain high crystallinity after immersing into water or removing the guest molecules during activation.

**Fig. 1:**
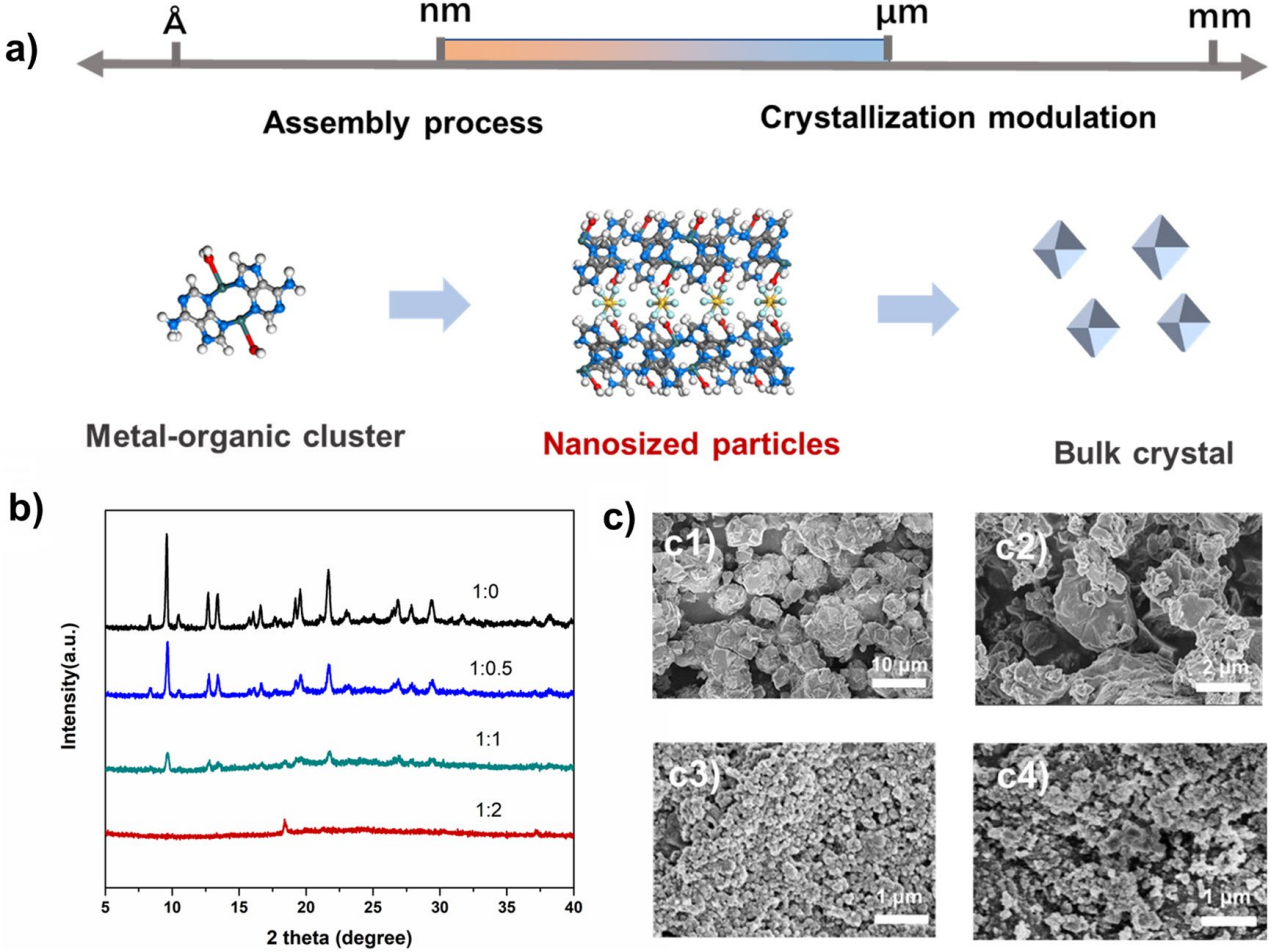
**a** Schematic of HOF-21 frame construction. **b** XRD patterns of HOF-21 prepared by different ratios of (NH4)_2_ SiF_6_/diethylamine. **c** SEM images of HOF-21m (**c1**) and HOF-21n with different ratios of (NH4)_2_ SiF_6_/diethylamine (**c2** for the ratio of 1:0.5, **c3** for the ratio of 1:1, **c4** for the ratio of 1:2)

Developing size-controlled synthesis of HOF materials is an important issue for the compatibility with the polymer and the final performance of the MMMs. In most cases, the size of particles is simultaneously affected by the reaction process and the crystallization process. Considering the Cu(ade)_2_ complexes were dispersed in solution at the molecular level, the hydrogen-bonded assembly of complexes with ${\text{SiF}}_{6}^{2 - }$ was vital to particle precipitation. Indeed, ${\text{SiF}}_{6}^{2 - }$, as a multi-armed reactive monomer, could hydrogen-bond with the amino on ade and bound water on copper ions, achieving the framework formation and crystal growth simultaneously. Therefore, during the hydrogen-bonded assembly process, a certain amount of diethylamine modulator was added to slow down the dissociation process of (NH_4_)_2_ SiF_6_ and the ${\text{SiF}}_{6}^{2 - }$ reactivity. The diethylamine also had electrostatic interaction with the HOF-21 particles to inhibit Ostwald ripening to some extent. Based on the reported stationary growth method, HOF-21m was prepared with the size of 5–50 um. The addition of modulator reduced the particle size to 50–200 nm. The molar ratio of diethylamine/(NH_4_)_2_ SiF_6_ affected both particle size and crystallinity, as shown in Fig. 1b, c. When the ratio of 1:2, the function of ${\text{SiF}}_{6}^{2 - }$ was completely inhibited and the particles were amorphous. Balancing particle size and crystallinity, the optimal ratio was 1:1 and the resultant HOF-21n were introduced as the filler to fabricate the MMMs.

### Structural Analysis of HOF-21

**Fig. 2:**
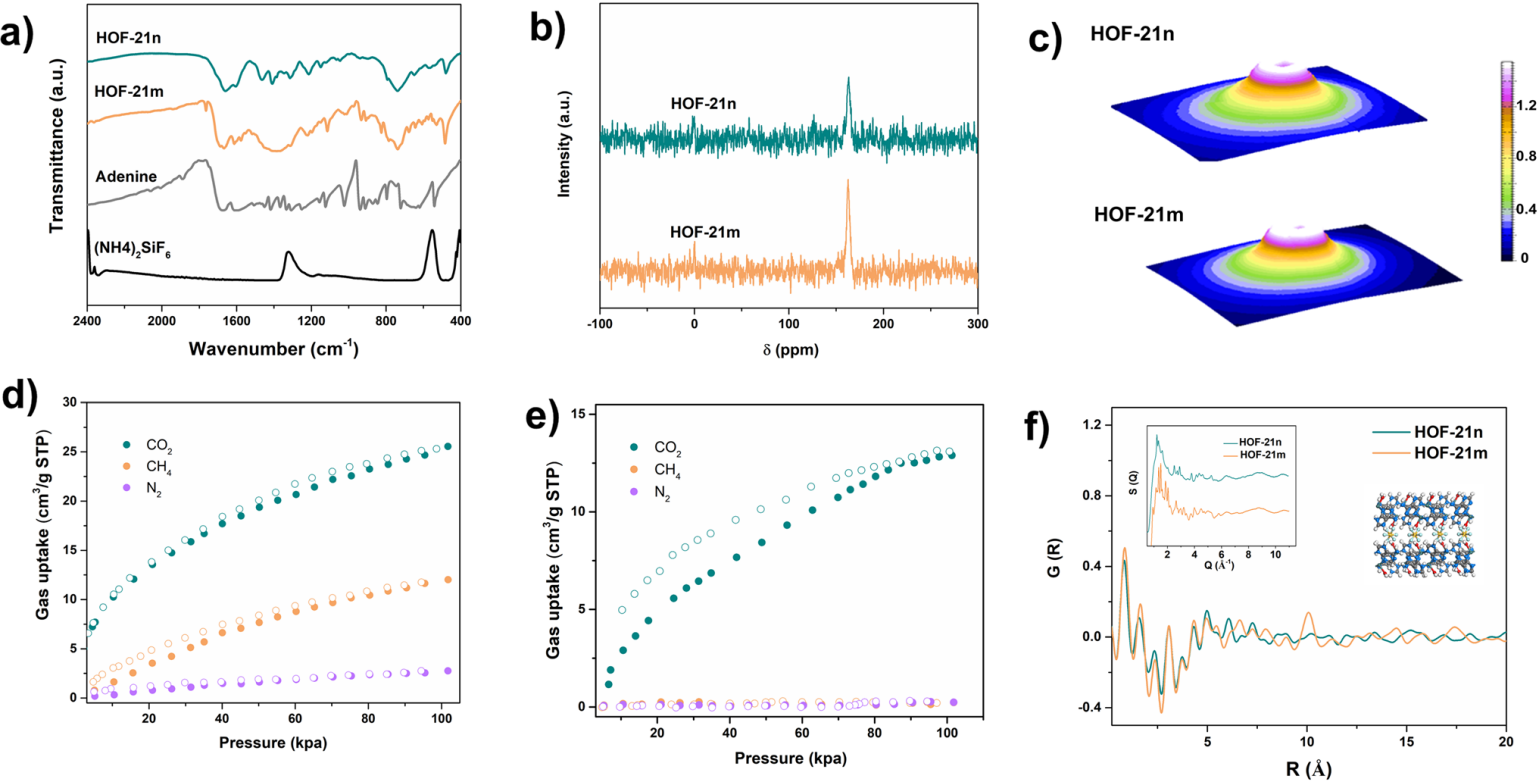
**a** FTIR spectrum of ade, (NH_4_)_2_ SiF_6_, HOF-21n and HOF-21m. **b** Solid-state NMR of HOF-21n and HOF-21m. **c** 3D-images of X-ray total scattering for HOF-21n and HOF-21m. **d** Gas uptakes of HOF-21m at 298 K.** e** Gas uptakes of HOF-21n at 298 K. **f** Pair function analysis of HOF-21n and HOF-21m, calculated from the data of **2c**. The inset is structural factor of HOF-21n and HOF-21m

The chemical compositions, structural properties and adsorption capacity of HOFm and HOF-21n were characterized and analyzed. As shown in Fig. 2a, the FTIR patterns of HOF-21m and HOF-21n displayed the compliance with ade. The peak at 480 cm^−1^ indicated the coordination bond of Cu–N. According to the pattern of C^13^ solid-state NMR, the peak of 175 ppm indicated the C of ade. No obvious peak of diethylamine confirmed that diethylamine only participated in the regulation of crystallization process, but did not act as the monomer embedded into the framework. The porosity of HOF-21m and HOF-21n was evaluated via the adsorption of CO_2_, CH_4_ and N_2_ (Fig. 2d, e). The gas uptakes of HOF-21m was 28 cm^3^ g^−1^ (CO_2_), 10 cm^3^ g^−1^ (CH_4_) and 2 cm^3^ g^−1^ (N_2_), respectively. In contrast, HOF-21n showed the CO_2_ uptake of 12 cm^3^ g^−1^ and negligible uptakes of CH_4_ and N_2_. HOF-21n showed the obvious decrease of CO_2_ adsorption, due to the relatively low degree of crystallinity and smaller particle size. Due to the strong interaction of metal ions and amino groups with acidic CO_2_, both the HOF-21m and HOF-21n showed higher affinity to CO_2_ than CH_4_ and N_2_. Based on the GCMC simulation of the CO_2_ adsorption data, the pore size of HOF-21m and HOF-21n both focused on 3.5 and 6.5 Å (Fig. S3). The pore distribution of HOF-21m was narrower than HOF-21n. The structural properties of HOF-21 in the short range were tested via synchrotron X-ray total scattering diffraction measurements. Figure 2c showed the 3D images of XRD patterns, thereby extracting the structural factor (*S*q) and the pair distribution functions (PDFs). The *G*(r) curves provided the information of the atom–atom distance and related bond information (Fig. 2f). The peaks at 0.9, 1.6, 2.3, 3, 5, 7.3, and 10 Å corresponded to the distance of C–C/C–N, Cu–N_1_, N–N, N–F, Cu–Cu_1_, Cu–N_2_, and Cu–Cu_2_, respectively. Due to the structure complexity of HOF-21, the atom–atom distances may overlap. Even so, the HOF-21m and HOF-21n were highly identical for distances up to 8 Å within the short range. In the medium range, the HOF -21m displayed more pronounced periodicity than that of HOF-21n. The thermal stability was confirmed from the thermogravimetric analysis curve (Fig. S4). HOFm and HOFn were followed a similar trend of mass-loss change, with the solvent removal process at 100 °C, bound water departure process at 100–200 °C and thermal decomposition process at 300 °C.

### Fabrication and Characterization of HOF-21-Based MMMs

**Fig. 3:**
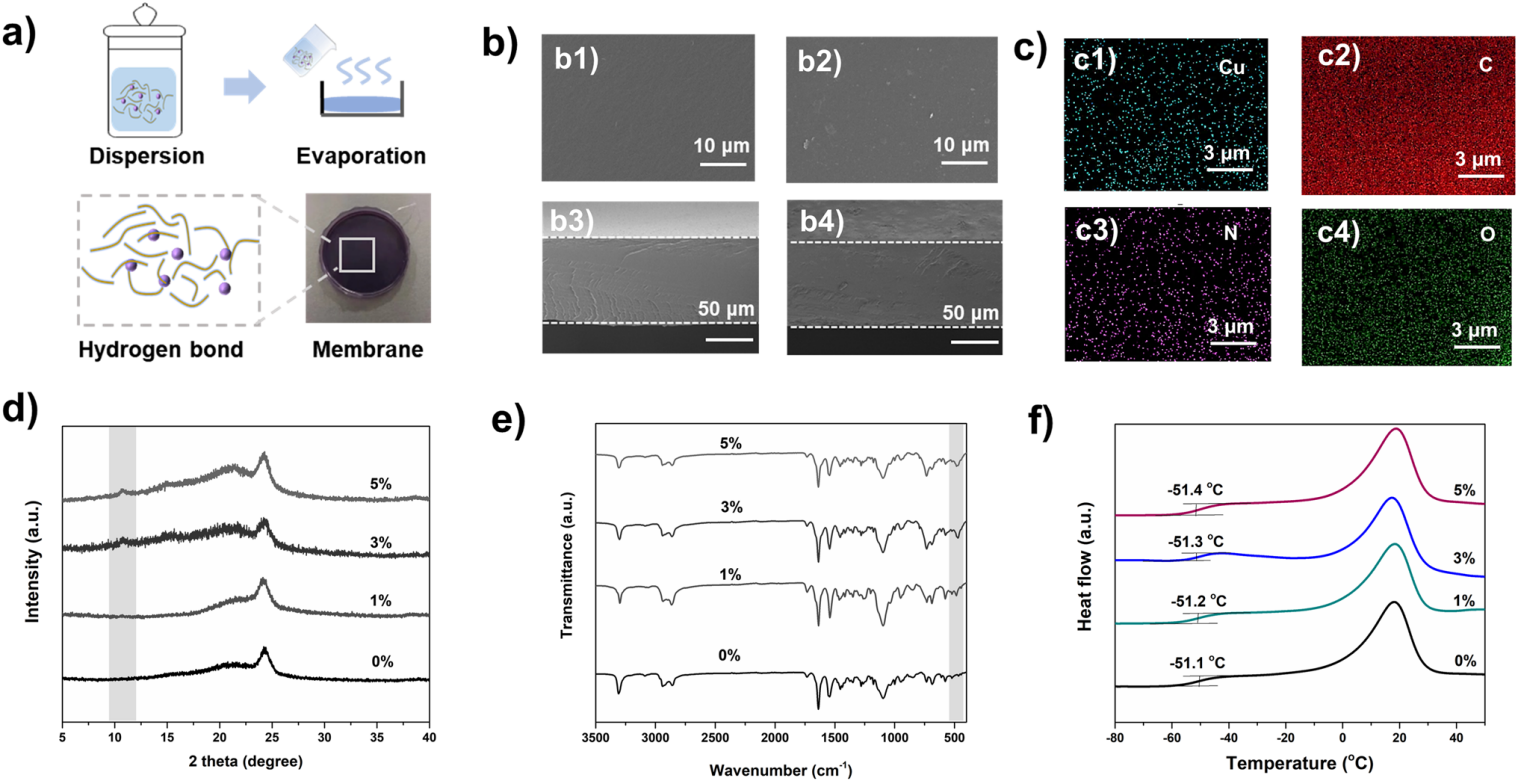
**a** Schematic of membrane construction. **b** Top-section SEM images of Pebax (**b1**) and MMMs (**b2**), the cross section membrane SEM images of Pebax (**b3**) and MMMs (**b4**). **c** EDS mapping of MMM (**c1**for Cu, **c2** for C, **c3** for N, **c4** for O). **d** XRD pattern, **e** FTIR spectra and **f** DSC analysis of the MMMs with the filler content of 0, 1, 3, and 5 wt%

The HOF-21-based membranes were fabricated via physical mixing of HOF-21 fillers and Pebax1657 polymer, followed by the solvent volatilization [26, 27]. The fillers were added in the form of colloidal solution, in case the dry samples difficultly dispersed evenly. HOF-21m and HOF-21n were added to the polymers, respectively. The cracks were observed in HOF-21m@ Pebax (Fig. S5). While HOF-21n@ Pebax was free of defects, indicative of better compatibility with polymers. The EDS mapping of HOF-21n@ Pebax MMMs further confirmed the uniform distribution of HOF-21n via detecting characteristic Cu element. To explore the optimal HOF-21n filler content, HOF-21n@ Pebax MMMs with different nanoparticles content ratios (0, 1, 3, and 5 wt%) were prepared and characterized. As the XRD patterns shown (Fig. 3d), the characteristic peaks of Pebax at 24°, corresponding chain spacing of 0.37 nm based on the Bragg formula. Due to the low filler content of 1%, the characteristic peak signal of HOF-21 is masked, and while a weak characteristic peak of 10° could be observed at the filler content of 3% and 5%. The chemical component of membranes was characterized by the ATR-IR (Fig. 3e). For the pristine Pebax membrane, the peak at 1094 cm^− 1^ corresponded the C–O–C stretching vibrations in poly(ethylene oxide) (PEO) block and the peaks at 3311, 1730, and 1638 cm^−1^ corresponded to N–H group, O–C=O group, and H–N–C=O group in polyamide (PA) block, respectively. Compared with the pristine Pebax membrane, the additional peaks at 480 cm^−1^ were observed in the HOF-21n @ Pebax MMMs and corresponded to the Cu–N bonds in the HOF-21. Other than that, no new chemical bonds were observed, indicative of hydrogen-bond interaction between the Pebax and the edge-amino of ade. The thermodynamic properties of MMMs were evaluated by the DSC. As shown in Fig. 3f, the glass transition temperature (*T*_g_) of pristine Pebax membrane was − 51.1 °C and the *T*_g_ of MMMs changed negligibly, further confirming the weak interaction between filler and Pebax matrix.

### Separation Performance of HOF-21-Based MMMs

**Fig. 4:**
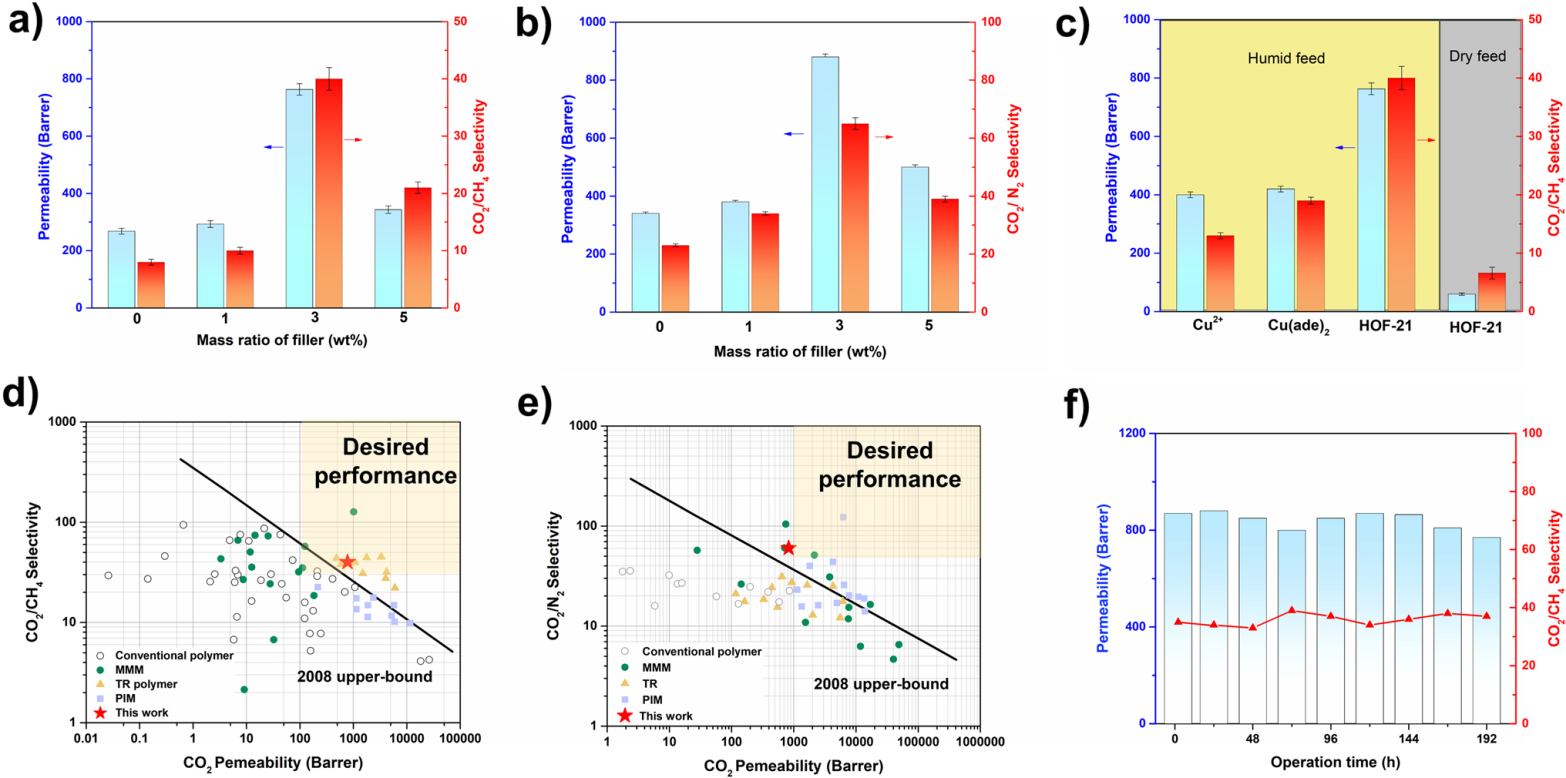
Separation performance of **a** CO_2_/CH_4_ and **b** CO_2_/N_2_ mixed gas separation performance of MMMs with different filler loadings. **c** Separation performance of MMMs with different fillers. The comparison of different membranes with best performance separation of the **d** CO_2_/CH_4_ and **e** CO_2_/N_2._ The data points extracted from reported literature [28–30]. **f** Long-term operation stability for CO_2_/CH_4_

The separation performance of the membranes was evaluated by the Wicke–Kallenbach method. Under the humid condition, the pristine Pebax membrane followed the solution-diffusion mechanism, while the MMMs followed both the solution-diffusion mechanism and the facilitated transport mechanism, corresponding to the mass transfer channels provided by polymers and HOF-21. The membranes with HOF-21m@ Pebax were non-selective due to the numerous defects. In contrast, the membranes with HOF-21n@ Pebax showed much superior performance. For the separation of CO_2_/CH_4_ (Fig. 4a), the pristine Pebax membrane showed the flux permeation of 240 Barrer and the selectivity of 8. With increasing the fillers content, membrane separation performance first increased and then decreased. The optimum membrane separation performance of HOF-21@ Pebax-3 showed the permeation flux of 780 Barrer and the selectivity of nearly 40. The addition of fillers could optimize membrane separation performance, but the excessive content would aggravate the agglomeration effect of particles and thus deteriorate the effect of fillers. For the separation of CO_2_/N_2_ (Fig. 4b), the MMMs exhibited the similar trends and the optimum membrane separation performance of HOF-21@ Pebax-3 showed the flux permeation of 840 Barrer and the selectivity of nearly 60.

To further investigate the facilitated transport effect of HOF-21, equimolar Cu^2+^, Cu(ade)_2_ and HOF-21n were used as fillers for comparative analysis (Fig. 4c). Compared with the pristine Pebax membrane, Cu^2+^ improved the membrane separation performance with the permeation of 400 Barrer and the selectivity of nearly 15. The Cu(ade)_2_ complexes showed significant enhancement in the selectivity, compared with Cu^2+^. HOF-21 achieved the improvement both in selectivity and permeability, with the aid of multiple interactions and frame channel. Cu^2+^, bound water and hydrogen bond network endowed HOF-21 with an efficient mass transfer process for CO_2_, similar to carbonic anhydrase (Fig. S8a). Introducing HOF-21 as the filler, the membrane is endowed with more channels to achieve high permeability. Meanwhile, the suitable channel size of 0.35 nm and multiple interactions between active sites and CO_2_ achieved a facilitated transport effect, leading to higher selectivity.

The transport process and relative energy change (Δ*E*) are fitted by simulation calculations as shown in Fig. S8b. In brief, CO_2_ first bound to the active site of Cu(ade)_2_ complexes in HOF-21, leading to a nucleophilic attack by the Cu-bound hydroxy ion onto the CO_2_ with Δ*E* of 2.71 eV. Then, ${\text{HCO}}_{3}^{ - }$ formed with Δ*E* of 1.43 eV and subsequently displaced by the water molecule inflowing through HOF-21 with Δ*E* of 1.28 eV. The ${\text{HCO}}_{3}^{ - }$ molecule likely binds to Cu^2+^ ion in a monodentate mode and its OH group is held at the Cu^2+^ ion due to the hydrogen bonding with the adjacent amino group of ade. The binding configuration leads to a metastable state and weak interaction, thereby greatly facilitating CO_2_ transport.

The effect of feed pressure was also investigated for CO_2_/CH_4_ separation (Fig. S9). With the pressure increasing from 2 to 5 bar, the permeability of HOF-21@ Pebax-3 showed a slight decrease from 763 to 707 Barrer along with the selectivity decrease from 39 to 31. The phenomenon was consistent with the previously reported facilitated transport membranes, lack of adequate carriers to transport CO_2_ under higher pressure. To evaluate the stability, a long-term gas permeation measurement of HOF-21@ Pebax-3 was carried out. The separation performance could maintain stable with the permeability above 700 Barrer and the selectivity above 35. Such separation performance exceeded the corresponding 2008 Robeson upper bound, superior to most CO_2_ separation membranes.

## Conclusions

In summary, we explored the potential of HOF-based mixed-matrix membranes in carbon capture for the first time. The HOF-21 nanoparticles with tunable size and high crystallinity were synthesized and had good compatibility with the bulk polymer. Incorporation of HOF-21 nanoparticles endowed the mixed-matrix membrane with more mass transfer channels and chemical functionality. The co-existence of Cu^2+^, bound water and hydrogen bond network jointly contributed to the conversion of ${\text{HCO}}_{3}^{ - } /{\text{CO}}_{2}$ and thus conferred facilitated transport mechanism. The HOF-based mixed-matrix membranes exhibited a permeability above 750 Barrer, a selectivity of ~ 40 for CO_2_/CH_4_ and ~ 60 for CO_2_/N_2_, surpassing the 2008 Robeson upper bound. This study demonstrates the great potential of HOF as versatile filler in mixed-matrix membranes and as the bulk membrane material in carbon capture.

### Supplementary Information

Below is the link to the electronic supplementary material.

Supplementary file1 (PDF 661 KB)
