# Supplementary material for: Engineering HOF-Based Mixed-Matrix Membranes for Efficient CO2 Separation
Source: Nanomicro Lett. 2023 Feb 14;15:50. doi: 10.1007/s40820-023-01020-w (PMC9929012; doi:10.1007/s40820-023-01020-w)
Supplement: Supplementary file 1 — Supplementary file1 (PDF 661 KB) [file 40820_2023_1020_MOESM1_ESM.pdf]

Supporting Information for

Engineering HOF-Based Mixed-Matrix Membranes for Efficient CO_2 Separation

Yuhan Wang[1,2,3], Yanxiong Ren[1,2,3], Yu Cao[1,2], Xu Liang[1,2], Guangwei He[1,2,3]

Hanze Ma[1,2,3], Hongliang Dong[4], Xiao Fang[1], Fusheng Pan[1,2,3,*], Zhongyi Jiang[1,2,3,*]

[1] Key Laboratory for Green Chemical Technology of Ministry of Education, School of Chemical Engineering and Technology, Tianjin University, Tianjin 300350, P. R. China

[2] Haihe Laboratory of Sustainable Chemical Transformations, Tianjin 300192 (China)

[3] Guangdong Laboratory of Chemistry and Fine Chemical Industry Jieyang Center, Guangdong Province 522000, P. R. China

[4] Center for High Pressure Science and Technology Advanced Research, Pudong, Shanghai 201203, P. R. China

*Corresponding authors. E-mail: fspan@tju.edu.cn (Fusheng Pan), zhyjiang@tju.edu.cn (Zhongyi Jiang)

Supplementary Figures

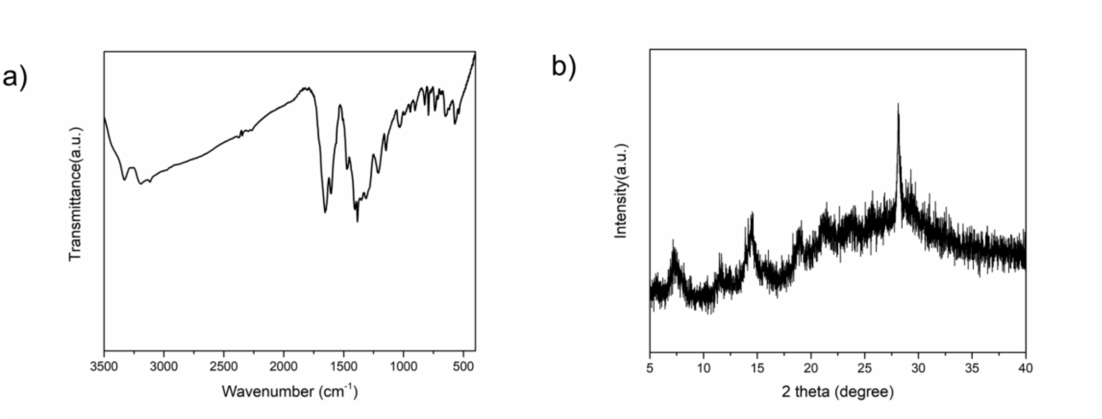

Fig. S1 FTIR spectra and XRD pattern of Cu (ade)$_2$

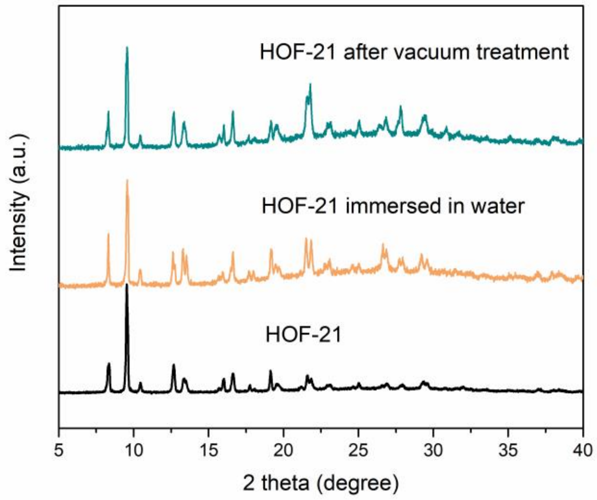

Fig. S2 XRD patterns of initial HOF-21 sample (a) and the HOF-21 samples after immsered in water and vacuum treatment

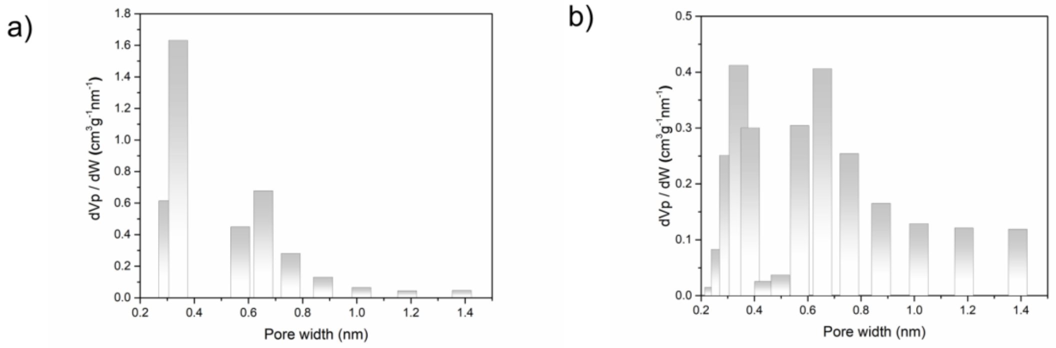

Fig. S3 Pore size distribution of HOFm (**a**) and HOFn (**b**)

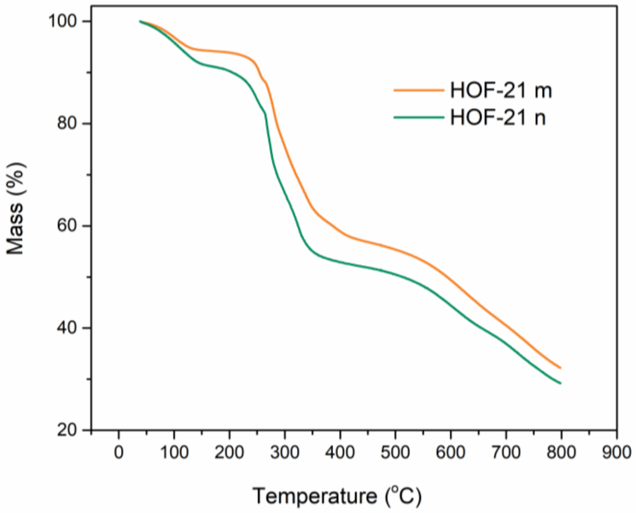

Fig. S4 Thermogravimetric analysis of HOF-21m and HOF-21n

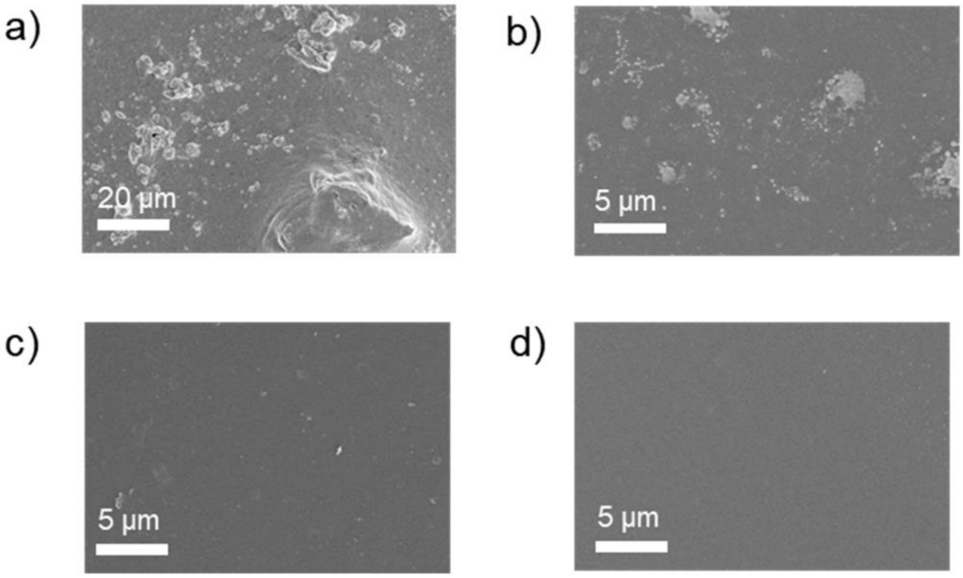

Fig. S5 SEM images of the membranes with the fillers regulated by the ratios of $(NH4)_2$ SiF_6/ diethylamine (**a** for the ratio of 1:0, **b** for the ratio of 1:0.5, **c** for the ratio of 1:1, **d** for the ratio of 1:2)

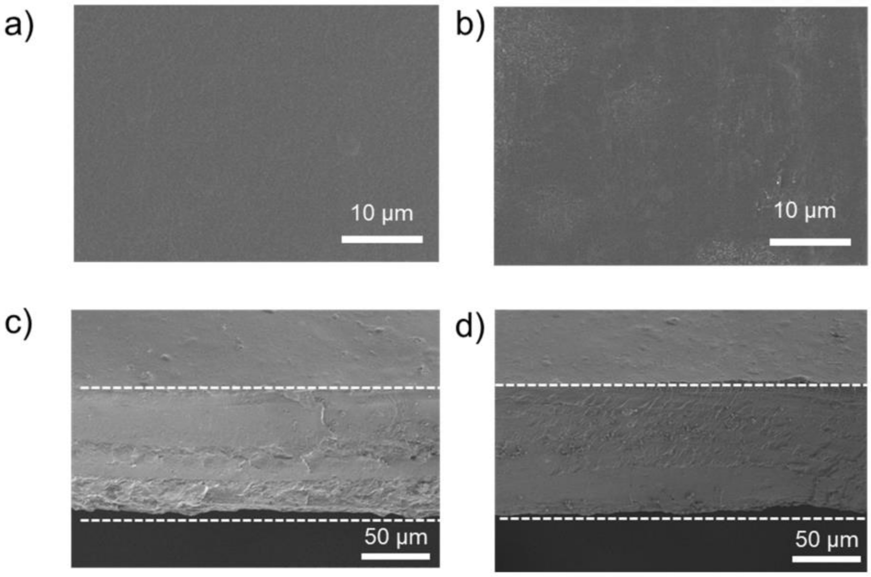

Fig. S6 The top-section SEM images of MMMs with the mass loadings of 1wt% (**a**) and 5 wt% (**b**), the cross-section membrane SEM images of MMMs with the mass loadings of 1wt% (**c**) and 5 wt% (**d**)

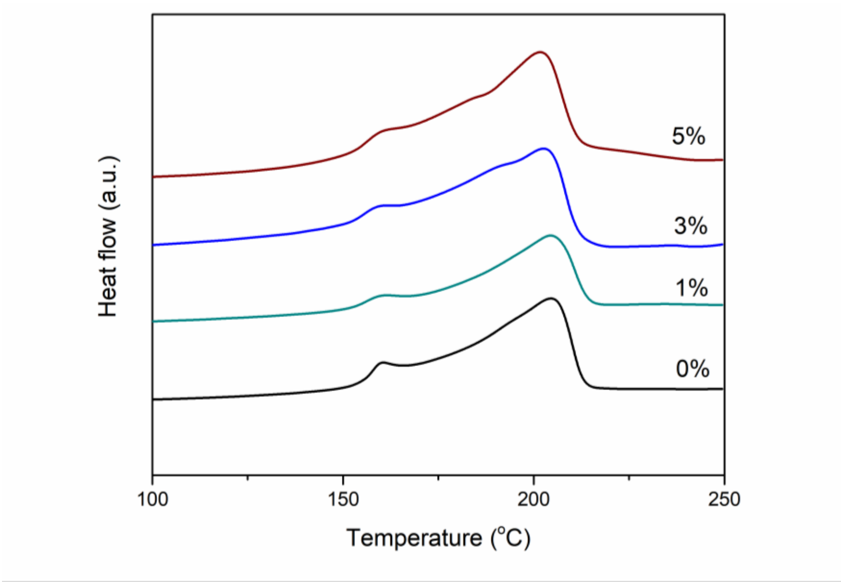

Fig. S7 DSC analysis of the MMMs with the filler content of 0, 1, 3, 5 wt%

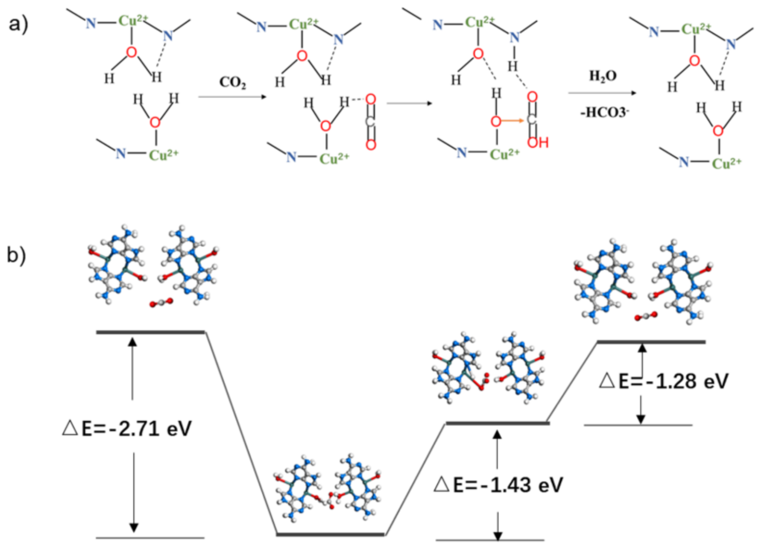

Fig. S8 Facilitated transport mechanism (**a**) and energy calculation (**b**)

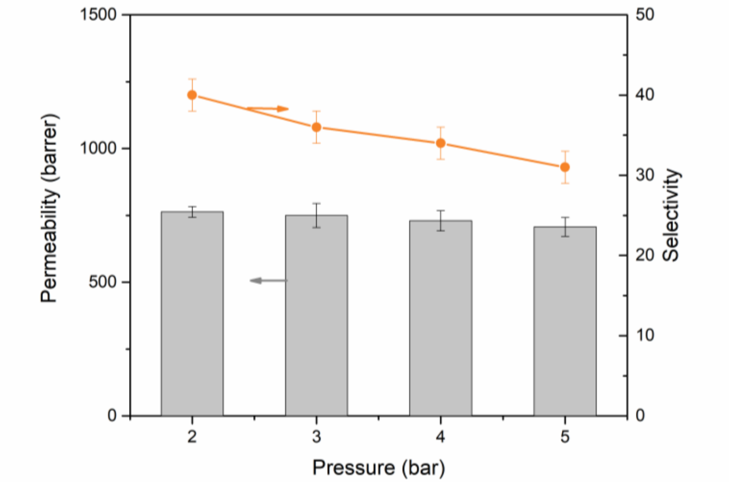

Fig. S9 Effect of pressure on the separation performance of HOF-21@ Pebax-3
